# Effect of a High-Protein High-Fibre Nutritional Supplement on Lipid Profile in Overweight/Obese Adults with Type 2 Diabetes Mellitus: A 24-Week Randomized Controlled Trial

**DOI:** 10.1155/2021/6634225

**Published:** 2021-04-15

**Authors:** Rachana Bhoite, Anitha Chandrasekaran, Varalakshmi Lalithya Pratti, Vinita Satyavrat, Shivani Aacharya, Amey Mane, Suyog Mehta, Ravindra Machhindra Kale, Gayathri Nagamuthu, Sasikala Selvaraj, Gayathri Rajagopal, Sudha Vasudevan, Shobana Shanmugam, Anjana Ranjit Mohan, Ranjit Unnikrishnan, Kamala Krishnaswamy, Viswanathan Mohan

**Affiliations:** ^1^Dr. Reddy's Laboratories Pvt Ltd., Ameerpet, Hyderabad, India; ^2^Department of Diabetology, Dr. Mohan's Diabetes Specialities Centre, Chennai, India; ^3^Department of Foods, Nutrition & Dietetics Research, Madras Diabetes Research Foundation, Chennai, India

## Abstract

**Background:**

Foods rich in protein and dietary fibre could potentially improve lipid profile in overweight or obese diabetic patients with dyslipidemia and, thereby, mitigate their risk of cardiovascular disease (CVD). In this study, the effect of providing high-protein high-fibre (HPHF) nutritional supplement in addition to standard care of type 2 diabetes mellitus (T2DM) on lipid profile was evaluated.

**Methods:**

In this open-label, parallel-arm, prospective, randomized study, a total of 100 overweight/obese participants with T2DM were randomized to either an intervention group (25 g HPHF nutritional supplement given twice daily along with a standard care of T2DM) or a control group (standard care of T2DM) for 24 weeks. Change from baseline in lipid parameters such as total cholesterol (TChol), high-density lipoprotein cholesterol (HDL-C), low-density lipoprotein cholesterol (LDL-C), and triglycerides (TG) was assessed between the intervention and control group at week 12 and week 24. Participant compliance was assessed using the dietary 24-hour recall. Statistical analysis was performed to assess the main effects on within- and between-group changes from baseline to end of 24 weeks.

**Results:**

Participants in the HPHF nutritional supplement group showed a statistically significant improvement in HDL-C levels by the end of 24 weeks (*p*=0.04) and a significant increase in protein and total dietary fibre intake (*p*=0.002 and *p*=0.00, respectively) compared to the control group. The TChol/HDL-C ratio was significantly lower (*p*=0.03) in the HPHF group from baseline to 24 weeks.

**Conclusion:**

Twice-daily consumption of a HPHF nutritional supplement significantly improved HDL-C levels. Inclusion of the HPHF supplement would be a useful effective aid for managing dyslipidemia in overweight/obese individuals with T2DM.

## 1. Introduction

Overweight and obesity have reached epidemic levels globally and their established comorbidities such as diabetes, dyslipidemia, hypertension, and cardiovascular disease (CVD) resulting in excess burden on healthcare resources in both developed and developing countries [1]. Prevalence of CVD is higher in adults with diabetes [2]. The risk of CVD increases with rising fasting plasma glucose levels [3] and is the major cause of death and disability among people with diabetes [4]. Being closely linked to the pathophysiology of CVD, dyslipidemia is a key independent modifiable risk factor for CVD [5]. Approximately 578 million people aged 20–79 years are expected to suffer with diabetes by 2030 across the globe [6]. About 72 to 85% patients with diabetes have dyslipidemia [7]. Type 2 diabetes mellitus (T2DM) is associated with reduction in life expectancy by as much as 10 years [4].

Many studies suggest nutrient composition of dietary strategies may be important and affect glucose and lipid profile in diabetes patients. Although an optimal diet for diabetes patients is unknown, currently evolving evidence suggests that high-protein [8] and high-fibre diets are beneficial. Replacement of animal-derived protein with vegetable-based protein may have positive effects on T2DM patients [9]. Vegetarian/plant-based diets can enhance weight loss and glycemic control and reduce cardiovascular disease risk factors due to their high fibre content, as they can have an effect on modulation of gut hormones and decrease inflammation. A large number of observational studies have documented the potential beneficial and health-promoting effects of plant-based diets in prevention and treatment of certain diseases (obesity, diabetes mellitus, dyslipidemia, atherosclerosis, CVD, cancer, and few gastrointestinal disorders) and, thus, promote healthy living [10].

It is widely acknowledged that addition of soy in diet of diabetes patients has multiple health benefits [11] such as a favorable effect on cholesterol and blood pressure. Soy consumption is associated with improvement in serum lipids as it decreases the micellar content and absorption of lipids through a combination of fibre, isoflavones, and phytoestrogens [12]. Even though the mechanism responsible for effects of proteins on plasma cholesterol has not been established clearly, it can be hypothesized that proteins with higher content of phosphorylated amino acids interfere with bile acid reabsorption or the amino acid content of the protein affects cholesterol absorption, tissue storage, synthesis, and excretion. Dietary protein may also alter cholesterol metabolism by affecting plasma hormones such as insulin, glucagon, thyroid hormones, and gastrointestinal inhibitory polypeptide [13]. Several studies suggested that soy protein intake is effective in reducing total cholesterol (TC), low-density lipoprotein cholesterol (LDL-C), and triacylglycerols and increasing high-density lipoprotein cholesterol (HDL-C) [14]. The study conducted by Anderson et al. found that soy protein intake is effective in reducing TC by 9.3%, LDL-C by 12.9%, and triacylglycerols by 10.5% and in increasing HDL-C by 2.4%. The American Diabetes Association (ADA) has recommended to include 26–50 g of soy protein per day (in place of animal protein), 25–30 g soluble fibre per day, Omega-3 fatty acids, and antioxidants (vitamin E, vitamin C, and *β*-carotene) in daily diet for the prevention and treatment of CVD [15, 16]. Studies on Asian Indians reported that soy supplementation in diet helps to manage blood glucose levels in both normal and overweight/obese patients with T2DM [17–19].

Consumption of dietary fibre delays gastric emptying and increases bulk-forming activity and, thus, provides a feeling of satiety, as well as viscosity-induced reduced absorption of cholesterol resulting in lower LDL-C concentration. Dietary fibre increases fecal excretion of bile acid and reduces its reabsorption in the small intestine. A decreased enterohepatic pool of bile acid upregulates the rate-limiting enzyme required for bile acid production (CYP7A1) which, in turn, promotes hepatic uptake of LDL-C from blood via upregulation of LDL receptor and CYP51. Dietary fibre fermentation produces short-chain fatty acids (SCFA) in the intestine which stimulate release of peptide YY (PYY) and glucagon-like peptide 1 (GLP-1) which help in decreasing LDL-C concentration. Moreover, dietary fibres, due to their viscosity, delay intestinal absorption of glucose which decreases insulin secretion. Lower insulin results in lower HMG-CoA reductase (*β*-hydroxy *β*-methylglutaryl-CoA reductase) which contributes to lower LDL-C concentration [20].

Sufficient evidence in terms of epidemiological and cohort studies are available that clearly shows that HDL-C levels are inversely associated with the risk of CVD [21]. Due to excess fat deposits in obesity, adipose cells bind to HDL-C [22], and the increased body fat may lead to an increased uptake of HDL particles from circulation resulting in reduction of plasma HDL levels. Low HDL-C is associated with increased risk for atherogenesis [23], and high HDL-C has been associated with decreased risk of coronary artery disease (CAD) [24] and has a cardio protective role due to its antioxidant activity, [25] possibly due to its role in the reverse cholesterol transport process where cholesterol in peripheral tissues is transported to the liver for reuse for bile acid synthesis, preventing its accumulation in the peripheral tissues and blood vessels [26].

Indian diet is predominant in refined cereal grains and also low in proteins [27]. Medical intervention, dietary modifications (high protein, fibre, mono and polyunsaturated fatty acids), and physical activity may help in managing lifestyle diseases [28–30]. The 2020 Indian Council of Medical Research (ICMR) committee report on estimated average requirement (EAR) for Indians [31] highlights the importance of increasing protein and fibre in Indian diets.

With this background, we designed a parallel-arm, randomized controlled trial to test the effect of a High-Protein High-Fibre (HPHF) supplementation on lipid profile, glycaemia (24-hour blood glucose control), nutrient intake, and body weight in adults with T2DM. The results on glycemic parameters were encouraging and have been published earlier [17]. This manuscript will focus on the effect of the nutritional supplement on lipid profile.

## 2. Materials and Methods

### 2.1. Study Participants

Participants in this parallel-arm, open-label, randomized controlled trial were identified from the medical records of a tertiary-care centre for diabetes in Chennai, India, based on prespecified eligibility criteria. Patients, aged 30–65 years of either gender, diagnosed with T2DM for at least 1-year duration and treated with stable doses of oral antidiabetic drugs for at least 3 months before screening were enrolled. Other key inclusion criteria were HbA1c between 7.0% to 12.0% and a body mass index (BMI) of ≥23 kg/m^2^ and <30 kg/m^2^ (Asian Indian cut off for overweight and obesity) [32].

Participants with T2DM who were on insulin injections or on unstable doses of oral hypoglycaemic agents (OHA) in the last 3 months were excluded from the study. Participants who had a history of acute infections in the last 1 month, respiratory disorders, eating disorders or lactose intolerance, history of hypoglycaemia in last 3 months, cancer or malignancy, heart attack, or stroke in the past 1 year were also excluded from the study. Additionally, pregnant and lactating women, participants who were on herbal or ayurvedic or traditional medicines that could affect blood glucose, and individuals who were planning to relocate within 2 years after study initiation or who had plans of longer duration of travel out of town were excluded from the study.

Initially, 320 overweight/obese participants with diabetes were identified from medical records. Out of them, 100 subjects were selected based on eligibility criteria. Participants were briefed by research dieticians about the study and the objectives. All participants underwent a run-in period in which 25 g of HPHF powder twice a day for one week was provided free of cost to assess compliance. Participants who completed a run-in period of 1 week and expressed their willingness to comply and take part in the study were randomized using computer-generated random numbers to either an intervention group (*n* = 50) or control group (*n* = 50). Baseline visit was completed one week after the run-in period.

Study protocol and informed consent form were reviewed and approved by the Independent Ethics Committee and the Institutional Review Board and registered in the Clinical Trial Registry of India CTRI/2018/04/012979. The study was conducted in accordance with the ethical principles of the Declaration of Helsinki and as per International Council for Harmonization and Good Clinical Practice guidelines. All participants provided written informed consent prior to study enrolment.

### 2.2. Dietary Intervention

Participants in the intervention group were instructed to consume one sachet of 25 g of HPHF nutritional supplement twice daily (one with breakfast and one in the evening) for 24 weeks along with standard care for diabetes. The participants were asked to mix 25 g of the HPHF nutritional supplement with ∼200 mL of water and consume without leftover. Participants in the control group were instructed to follow standard care of diabetes for 24 weeks. For both the groups, all dietary advice was individualized and provided by dieticians, as is the standard practice for the tertiary-care centre for diabetes in Chennai, India, where this study was conducted.

Participant compliance was assessed using the dietary 24-hour recall collected by trained dieticians in a face-to-face interview. The EpiNu nutrient database (Madras Diabetes Research Foundation, India) was used to assess the food and nutrient intake (mainly macronutrients) from the 24-hour dietary recalls. The average of 5 recalls collected during the 24-week intervention was compared to the baseline (3 days of dietary recall at baseline) in order to improve the precision and accuracy of the estimates of dietary intake during the intervention period.

The macro- and micronutrient breakdown of the HPHF nutritional supplement are shown in Table 1.

### 2.3. Outcome Assessments

Anthropometry: body weight (kg) (electronic OMRON machine; 171 Omron HBF 212, Tokyo, Japan), height (cm), and waist circumference were measured at baseline, monthly once, and at the end of the study as per standard protocols. The body mass index (BMI) was calculated as weight (kg) divided by height^2^ (meter^2^). In addition, a validated physical activity questionnaire was used to assess the physical activity level in the study participants both at baseline and at the end of the study duration.

Blood pressure: blood pressure was assessed twice on each occasion (baseline, every month, and end of study) at 5-minute intervals using an electronic OMRON machine (Omron HEM 7120, Tokyo, Japan). Participants were seated comfortably with the back straight and feet flat on the floor, and the average of the two readings taken after 10 min of rest was noted as the blood pressure.

Biochemical: blood samples (5 ml) were collected for assessment of fasting blood glucose, HbA1c, and lipid profile at baseline and at the end of 12 weeks and 24 weeks. Lipid profile (total cholesterol, HDL-C, and triglycerides) and plasma glucose were assessed in a laboratory certified by the National Accreditation Board for Testing and Calibration Laboratories and the College of American Pathologists on a Hitachi 912 Autoanalyzer (Hitachi, Mannheim, Germany). The Beckman Coulter AU 2700/480 Autoanalyzer (Beckman AU, Olympus, Ireland), was used to measure serum cholesterol (cholesterol esterase oxidase-peroxidase-amidopyrine method), serum triglycerides (glycerol phosphate oxidase-peroxidase-amidopyrine method), and HDL-C by the direct method with polyethylene-glycol-pretreated enzymes. LDL-C was calculated using the Fried Wald formula. The coefficients of variation for the biochemical assays ranged from 3.1 to 7.6%.

### 2.4. Compliance and Adverse Event Assessments

Participant's compliance was determined by (a) returning the empty sachets in the intervention group and (b) calculating the difference between nutrient intakes assessed using 24-hour recalls at baseline (3 days of 24 -hour recalls) and twice every month during the study period. An adverse-event questionnaire was collected at the end of every month to record if the participants in the intervention group experienced any discomfort including bloating, diarrhoea, or any abdominal discomfort. Adverse events, if any, were assessed and recorded, and appropriate medical intervention was given.

### 2.5. Statistical Analysis

Statistical analysis was performed using SAS 9.2 version (SAS Institute Inc., Cary, NC). Baseline demographics and clinical characteristics were estimated using independent *t*-tests for continuous variables and the Pearson chi-square test for categorical variables. A Generalized Linear Model (GLM) was used to assess the main effects on within and between changes in anthropometric, biochemical parameters, and dietary variables at different time points (baseline vs. end of 24 weeks), and covariates were adjusted as an effect of interaction between and within participants. The outcome variables were further adjusted for potential confounders as appropriate. A two-tailed *p* value <0.05 was considered for statistical significance.

## 3. Results

### 3.1. Demographics and Baseline Characteristics

**Figure 1:**
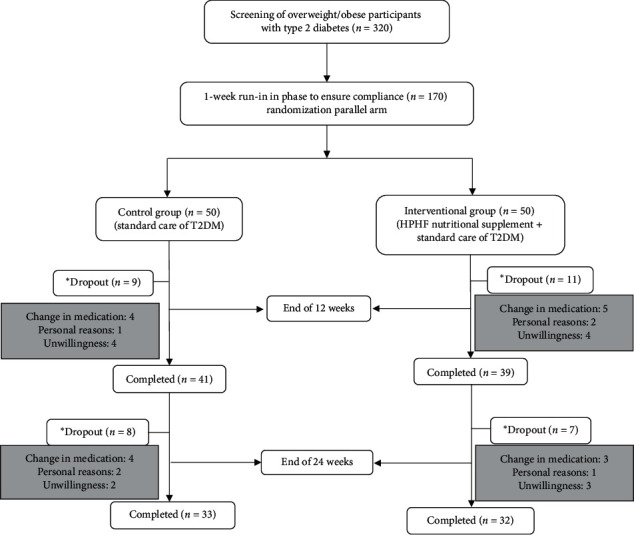
Study design. Note: a one-week run-in phase was performed before randomization to ensure compliance of participants enrolled in the study. Lipid profile was carried out at baseline (first 15 days), interim visit (15 days at the end of 12 weeks), and end of the study (15 days at the end of 24 weeks). For dietary compliance assessments, average of 5 recalls were collected during the 24 weeks' intervention and compared to baseline (3 days of dietary recall at baseline). *∗*Total dropouts (*n* = 35): reasons include lack of response or unwillingness to continue the study, personal reasons (such as out-of-station travel and family function), and change in diabetes medication.

Of 320 overweight/obese individuals with T2DM, 100 were enrolled and randomized to either the intervention group (HPHF nutritional supplement daily for 24 weeks with standard care of T2DM (*n* = 50)) or control group (standard care of T2DM (*n* = 50)) (Figure 1). Over the study duration of 24 weeks, 18 participants in the intervention group and 17 in the control group dropped out from the study due to various reasons such as unwillingness to continue the study, personal reasons including out-of-station travel and family functions, and change in diabetes medication in the control group. No one complained of any adverse event.

The mean age of the study participants at baseline in control and intervention groups were 50 and 51 years, respectively. Significant differences were observed in biochemical parameters such as HbA1C (*p*=0.04) and TChol/HDL-C ratio (*p*=0.007) and dietary nutrient intake of carbohydrates (*p*=0.01), proteins (*p*=0.01), and total calorie intake per day (*p*=0.01) at baseline between the groups. Other values were comparable across both groups at baseline (Table 2). There were no side effects of the nutritional intervention reported in the adverse event questionnaire.

### 3.2. Nutrient Intake

Control group showed a significant increase in total calories, fat (g/d and %E); monounsaturated fatty acids (MUFA) (g/d and %E), saturated fatty acids (SFA) (g/d and %E), and polyunsaturated fatty acids (PUFA) (g/d) and significant reduction in protein and carbohydrates (%E) at the end of the 24 weeks compared to baseline. The intervention group also showed the same trend and, in addition, there was a significant increase in protein (*p*=0.002) and dietary fibre (*p*=0.00) intake at the end of 24 weeks compared to the control group (Table 3).

### 3.3. Lipid Profile

A significant increase was noted in the intervention group for HDL-C levels (4 mg/dL, *p*=0.04) at the end of 24 weeks compared to the control group (Table 4). A significant decrease in the TChol/HDL-C ratio (*p*=0.03) was observed within the intervention group at the end of 24 weeks (Table 4). Even though not statistically significant, there was a modest decrease in other lipid parameters like LDL-C and TG levels in intervention group from baseline to week 24 (Table 4).

## 4. Discussion

The present study evaluated the effect of an HPHF nutritional supplement on lipid parameters in overweight/obese individuals with T2DM in India. The results demonstrated that twice-daily consumption of the HPHF nutritional supplement with standard care of diabetes in the intervention group showed a significant increase in protein and dietary fibre intake over a period of 24 weeks. Simultaneously, addition of the HPHF nutritional supplement in the intervention group led to a significant increase in HDL-C levels by the end of 24 weeks. Although reduction in the TChol/HDL ratio did not reach conventional levels of statistical significance in the group as a whole, within the intervention group, there was a statistically significant improvement at the end of 24 weeks. Even though reduction (LDL-C, TG, and TChol/HDL-ratio) and improvement (HDL-C) in lipid parameters are small, they are sufficient to result in metabolic changes that might be expected to translate into clinical benefits in reducing CVD risk.

HDL-C is involved in reverse cholesterol transport and helps in the movement of sterols from peripheral cells to the liver, and excess cholesterol from peripheral tissues is removed and delivered to liver which, in turn, is either redistributed to tissues or removed from body; thus, high levels of HDL-C can help reduce coronary heart disease (CHD) risk [33]. A meta-analysis of prospective studies revealed that, for every 1 mg/dL increase in HDL-C levels, the risk of CHD decreases by 2–3% [34]. According to Indian Heart Association, every 10-point increase in HDL-C may reduce the risk of heart disease by half [35]. A population-based study conducted by the Indian Council of Medical Research (ICMR) in 4 different regions of India reported that 72.3% of participants had low HDL-C levels, 29.5% had hypertriglyceridemia, and 12% had high LDL-C levels. Hence, low HDL-C was the most common lipid abnormality prevalent in Indians [36]. Several studies have documented the link between low HDL-C levels and risk of CVD. The Framingham heart study reported that, for every 1% decrease in HDL-C levels, the risk of CHD increases by 3% [37]. The Quebec Cardiovascular Study concluded that, for every 10% reduction in HDL-C, the risk of CAD increases by 13% [38].

Several studies have reported that Asian Indians are generally deficient in protein and fibre. The estimated average requirement (EAR) for protein in adults was approximately 0.66 g/kg/day, and the recommended dietary allowance (RDA) for fibre is between 25–52 g/day based on activity level and gender in the latest ICMR committee report (2020) for Indians [31]. As per the National Nutrition Monitoring Bureau (NNMB) study, Indian diets derive almost 60% of their protein from cereals with low digestibility and quality; this might have led to protein deficiency in certain sections of population [39]. On the other side, dietary fibre is not considered as a nutrient as there is no deficiency state; hence, an adequate intake (AI) level was set to provide its physiological benefits. In India, dietary fibre consumption varies among different socioeconomic groups from 15 to 41 g/day, and it is dependent on the type of food consumed based on the regional differences [40].

A systematic review on the effect of high- vs. low-protein diets suggested that even a moderate increase in protein content in diet is associated with favorable effects on HDL-C levels [41]. A study in US adults showed that an increase in dietary protein intake was associated with an increase in HDL-C levels and the potential health benefits of high-protein diets were more pronounced in overweight individuals than individuals with normal weight [42]. The mechanism by which protein is associated with upregulated HDL cholesterol production is not clearly understood; nevertheless, habitually consuming a higher-protein diet was associated with higher HDL-C levels irrespective of carbohydrate and fat intake, and the intrinsic properties of protein, unrelated to its energy content, may appear to be partially responsible for these effects ([42]).

Studies have reported a significant increase in HDL-C levels and decrease in TC, LDL-C, and TChol/HDL ratio with higher intake of dietary fibre [43–45]. Observational studies have shown that dietary fibre intake is associated with decreased risk of CVD [46]. The mechanisms by which increased dietary fibre intake reduces plasma cholesterol concentrations are unclear. However, increase in bile-acid excretion and reduction in cholesterol absorption may have contributed to this finding [47]; also, fibre intake changes intestinal motility and fibres with high viscosity delay absorption of macronutrients, leading to increased insulin sensitivity and increased satiety [48]. A study conducted in Asian Indian diabetic population showed that the individuals who consumed less than the population median dietary fibre intake of <29 g/day are at higher risk of developing CVD [49].

It should also be noted that several studies have highlighted the importance of consuming soy as a part of diet due to its protective effects against heart diseases, cancer, osteoporosis, hypertension, hyperglycemia, inflammation, and obesity [50, 51]. A meta-analysis that evaluated the association between soy and risk of CVD indicated that consumption of soy reduced risk of CVD, stroke, and CHD [52]. Another meta-analysis that examined the effect of soy and soy products found a lowering effect on TC, LDL-C, and TG, in addition to increased HDL-C among individuals with T2DM [53]. A wide range of studies suggest convincing evidence of soy protein in the cholesterol-lowering effect and soy products provide a large amount of protein with high-quality amino acids [54]. Based on the current evidence, the AHA nutrition committee recommended that daily consumption of soy protein can improve lipid profiles in hypercholesterolemic patients [16].

Considering the consequences of low protein and fibre intake on health outcomes, the HPHF nutritional supplement was designed with approximately 30% proteins (combination of milk and plant protein) and 12% fibre, with low glycemic index (GI) (approximately 27). As HDL-C is synthesized in the liver and intestines, we can hypothesize that the mechanism by which HPHF nutritional supplement increased HDL-C levels may be dependent on the scavenger receptor class B type 1 (SR-B1) pathway. After uptake of HDL-C by the SR-B1 pathway into the liver, the smaller HDL-C particles are released back into the circulation, thus increasing HDL-C concentration [55, 56]. Even though HDL-C has a role against CVD, nevertheless the TC/HDL-C ratio is more sensitive in protecting against morbidity and severity of CVD. Our study findings suggest that twice-daily administration of the HPHF nutritional supplement along with standard care of diabetes has beneficial effects in increasing HDL-C levels, assuming position in light of the fact that we currently do not have safe and effective pharmacotherapy for increasing HDL-C levels.

### 4.1. Strengths and Limitations

The present study provides robust data to support the clinical benefits of the HPHF nutritional supplement in overweight/obese Asian Indian patients with T2DM. Long-term intervention studies with nutritional supplements in diabetes patients are rare, and the strengths of our study include long intervention period of 24 weeks and acceptability of the HPHF nutritional supplement in the intervention group, as no adverse events were reported during the study. The primary limitation of the study is the sample size as the total number of subjects studied was relatively small. Hence, further studies with larger sample sizes are necessary to validate the impact of the HPHF nutritional supplement on clinical lipid profile.

## 5. Conclusions

Based on the evidence generated from this intervention trial, twice-daily administration of the HPHF nutritional supplement with standard care of diabetes for 24 weeks in overweight/obese adults with T2DM caused a significant increase in HDL-C levels and a modest decrease in LDL-C levels. A combination of the HPHF nutritional supplement with diabetic diet and other lifestyle changes could have a positive impact on controlling dyslipidemia which is a key independent modifiable risk factor of CVD.

## Figures and Tables

**Table 1:** Nutritional composition of the HPHF nutritional supplement.

Nutrients (unit)	Per 25 g	Per 100 g
Energy (kcal)	85.5	342
Protein (g)	7.5	30
Carbohydrates (g)	6.75	27
Added sugar (g)	0	0
Dietary fibre (g)	3	12
Fat (g)	2.5	10
Monounsaturated fatty acids (g)	1.625	6.5
Polyunsaturated fatty acids (g)	0.5	2
Saturated fatty acids (g)	0.375	1.5
Cholesterol (mg)	<0.25	<1
Trans fatty acids (g)	0	0

Vitamins		
Vitamin C (mg)	13.51	54.05
Vitamin B5 (mg)	1.11	4.45
Vitamin E (mg)	1.01	4.05
Vitamin B6 (mg)	0.27	1.08
Vitamin B2 (mg)	0.25	0.98
Vitamin B1 (mg)	0.19	0.76
Vitamin A (mcg)	60.81	243.24
Folic acid (mcg)	31.13	124.52
Vitamin K (mcg)	5.41	21.62
Biotin (mcg)	3.34	13.34
Vitamin D2 (mcg)	1.78	7.12
Vitamin B12 (mcg)	0.34	1.35

Minerals		
Potassium (mg)	222.36	889.43
Magnesium (mg)	17.79	71.15
Iron (mg)	1.34	5.34
Zinc (mg)	1.11	4.45
Manganese (mg)	0.31	1.25
Copper (mcg)	137.16	548.65
Iodine (mcg)	25.68	102.70
Chromium (mcg)	11.49	45.95

Ingredients: skimmed milk powder, defatted soya flour, gram flour, high oleic sunflower oil, fructo-oligosaccharides powder, corn dextrin, ragi flour, minerals premix, soya oil, sodium chloride, bulking agent (INS 466), emulsifier (INS 322 (i), INS 415), sweetener (INS 955), vitamin premix, anticaking agent (INS 551).

**Table 2:** Demographic and baseline characteristics of the study participants {intent to treat (*n* = 100)}.

Variables	Control group (*n* = 50)	Intervention group^$^ (*n* = 50)	*p* value*∗*#
	Mean ± SD	Mean ± SD	
Age (years)	50 ± 8	51 ± 8.2	0.51
Male *n* (%)	20 (40)	31 (62)	**0.04**
Height (cm)	158 ± 10	162 ± 8.3	0.08
Weight (kg)	73.3 ± 14.0	73.5 ± 14.2	0.92
Body mass index (kg/m^2^)	29.3 ± 5.4	28.0 ± 4.3	0.19
Waist circumference (cm)	98 ± 12	97 ± 11	0.57
Systolic blood pressure (mmHg)	126 ± 17	125 ± 15	0.90
Diastolic blood pressure (mmHg)	85 ± 10.8	83 ± 7	0.27
Fasting blood sugar (mg/dL)	163 ± 52	173 ± 51	0.31
HbA1c (%)	8.3 ± 1.3	8.9 ± 1.4	**0.04**
Triglycerides (mg/dL)^a^	140 (125, 159)	163 (140, 190)	0.10
Total cholesterol (mg/dL)	188 ± 41	195 ± 48	0.40
TChol/HDL-ratio	4.5 ± 1.0	5.0 ± 1.0	**0.007**
Duration of diabetes (years)	5.0 ± 2.7	5.1 ± 2.7	0.68
Energy (kcal/day)	1570 ± 345	1401 ± 336	**0.01**
Carbohydrate % E/day	58.1 ± 6.0	59.0 ± 5.7	0.43
Carbohydrate (g/day)	224.8 ± 46.2	202.9 ± 41.8	**0.01**
Protein % E/day	12.1 ± 1.1	12.0 ± 1.4	0.81
Protein (g/day)	47.0 ± 10.5	42.0 ± 9.6	**0.01**
Total fat (g/day)	44.7 ± 16.1	39.1 ± 18.6	0.10
Total fat % E/day	24.8 ± 5.0	24.0 ± 5.4	0.40
Monounsaturated fatty acids (g/day)	11.3 ± 4.6	10.0 ± 4.7	0.16
Monounsaturated fatty acids % E/day	6.3 ± 1.7	6.2 ± 1.5	0.71
Polyunsaturated fatty acids (g/day)	16.2 ± 6.1	14.4 ± 7.0	0.16
Polyunsaturated fatty acids % E/day	9.0 ± 1.8	8.8 ± 2.1	0.69
Saturated fatty acids (g/day)	12.4 ± 5.0	10.6 ± 4.7	0.06
Saturated fatty acids % E/day	6.9 ± 2.1	6.6 ± 2.0	0.37
Total dietary fibre (g/day)	25.5 ± 7.0	23.8 ± 7.3	0.24
Physical activity level	1.4 ± 0.1	1.5 ± 0.2	0.34

Data presented as mean ± SD. ^a^Antilogarithmic values were presented as mean and 95% CI. ^$^High-Protein High-Fibre 2 sachets per day (25 g/sachet in 200 ml water) for 24 weeks. *∗*Significance tested using an independent *t*-test, #significance tested using the chi-square test.

**Table 3:** Change in nutrient intake of control and intervention groups over 24 weeks (*n* = 65).

	Control group			Intervention group^$^			Between-group difference
	Baseline (*n* = 33)	24 weeks (*n* = 33)	*p* value^*∗*^	Baseline (*n* = 32)	24 weeks (*n* = 32)	*p* value^*∗*^	
Energy (kcal/day)	1549 ± 318	1660 ± 311	**0.01**	1418 ± 227	1543 ± 254	**0.001**	0.88
Carbohydrate % E	58.1 ± 4.5	55.7 ± 4.2	**0.002**	58.3 ± 4.3	55.0 ± 3.5	**0.001**	0.23
Carbohydrate (g)	223.2 ± 48.3	227.3 ± 39.4	0.48	204.2 ± 28.9	209.9 ± 29.6	0.32	0.86
Protein % E	12.4 ± 1.0	12.0 ± 0.9	**0.04**	12.6 ± 1.3	13.9 ± 1.2	**0.000**	**0.00**
Protein (g)	47.9 ± 10.9	50.4 ± 10.8	0.09	44.5 ± 8.3	52.9 ± 9.1	**0.000**	**0.002**
Total fat % E	24.4 ± 3.9	26.4 ± 3.9	**0.002**	24.0 ± 3.6	25.9 ± 3.5	**0.03**	0.88
Total fat (g)	43.0 ± 12.4	50.4 ± 14.5	**0.001**	39.0 ± 10.7	45.7 ± 13.9	**0.01**	0.65
Monounsaturated fatty acids % E	6.5 ± 1.3	7.0 ± 1.2	**0.01**	6.4 ± 1.0	7.2 ± 1.0	**0.004**	0.44
Monounsaturated fatty acids (g/day)	11.4 ± 3.6	13.3 ± 3.9	**0.004**	10.5 ± 2.8	12.6 ± 3.7	**0.002**	0.76
Polyunsaturated fatty acids % E	9.0 ± 1.3	9.2 ± 1.1	0.51	9.1 ± 1.4	9.6 ± 1.7	0.23	0.84
Polyunsaturated fatty acids (g/day)	15.9 ± 4.6	17.6 ± 4.9	**0.03**	14.6 ± 3.3	16.9 ± 6.2	0.06	0.96
Saturated fatty acids % E	6.9 ± 1.8	7.9 ± 1.9	**0.00**	6.4 ± 1.8	7.2 ± 1.5	**0.01**	0.51
Saturated fatty acids (g/day)	12.0 ± 4.0	15.0 ± 5.2	**0.00**	10.6 ± 4.3	12.7 ± 3.9	**0.001**	0.28
Total dietary fibre (g/day)	26.7 ± 6.5	24.9 ± 5.5	0.06	25.3 ± 5.8	28.9 ± 5.1	**0.000**	**0.00**

^*∗*^Data presented as mean ± SD. *Note*. ^$^High-Protein High-Fibre 2 sachets (25 g/sachet in 200 ml water) for 24 weeks. ^*∗*^*p* value <0.05 considered as significant using the paired *t*-test.

**Table 4:** Change in blood lipid levels of control and intervention groups over 24 weeks based on intent to treat (*n* = 65).

Variables	Control group			Intervention group^$^			Between-group *p* value*∗*
	Baseline (*n* = 33)	24 weeks (*n* = 33)	Within group *p* value	Baseline (*n* = 32)	24 weeks (*n* = 32)	Within group *p* value	
Triglycerides (mg/dL)^a^	142 (123–165)	130 (113–150)	0.30	157 (130–188)	152 (122–189)	0.81	0.81
Total cholesterol (mg/dL)	182 ± 41	179 ± 43	0.56	194 ± 46	194 ± 41	0.82	0.58
High-density lipoprotein cholesterol (mg/dL)	40 ± 8	42 ± 10	0.55	39 ± 10	43 ± 10	0.08	**0.04**
Low-density lipoprotein cholesterol (mg/dL)	111 ± 35	109 ± 37	0.64	123 ± 37	119 ± 36	0.39	0.81
TChol/HDL-ratio	4.6 ± 1	4.4 ± 1.0	0.27	5.0 ± 1.0	4.7 ± 0.9	**0.03**	0.07

*∗*Data presented as TChol/HDL ratio mean ± SD. ^a^Antilogarithmic values were presented as mean and 95% CL. Outcome adjusted for baseline (medication count per day), PAL: physical activity level (categorical), gender (category), carbohydrate (g/d), HbA1c (%), energy kcal/d (<,> median), and protein (g/d). ^*∗*^*p* value <0.05 considered as significant using the generalized linear model. ^$^High-Protein High-Fibre 2 sachets (25 g/sachet in 200 ml water) for 24 weeks.

## Data Availability

The clinical study data used to support the findings of this study are available from the corresponding author upon request.
